# Is labor force participation detrimental to the mental health of rural older adults? The mediating role of attitudes toward aging

**DOI:** 10.3389/fpubh.2025.1664235

**Published:** 2025-10-30

**Authors:** Yanjun Liu, Xiaoyuan Ji

**Affiliations:** School of Public Administration, Xi’an University of Architecture and Technology, Xi’an, China

**Keywords:** labor participation, aging attitudes, mental health, mediating effect, rural older adults

## Abstract

**Background:**

Population aging is a significant global issue. In China, the widening urban-rural disparity in population aging has contributed to problems such as the hollowing out of rural areas and a decline in the labor force. This situation, in turn, has subjected older adults in rural areas to significant psychological pressure. Thus their mental health has been widely emphasized. This study is dedicated to analyzing the linkages between mental health, attitudes toward aging, and their labor force participation among rural Chinese older adults in order to promote the construction of an age-friendly social system.

**Methods:**

Based on data from the 2020 China Longitudinal Aging Study (CLASS), this study selected rural household residents aged 60 and above as its research subjects. Correlation analysis examined the relationships among labor force participation, mental health among older adults, and attitudes toward aging. OLS regression model was used to investigate the impact of labor force participation on mental health. Stepwise regression and Bootstrap mediation analysis were applied to test the mediating role of attitudes toward aging. Propensity Score Matching was utilized to address endogeneity issues, ensuring the robustness of the findings.

**Results:**

(1) This study finds that labor participation significantly promotes the mental health of rural older adults, manifested explicitly in reduced depressive symptoms. However, this beneficial effect exhibits a threshold, resulting in a “U-shaped” impact where the influence of labor participation first increases and then decreases. (2) Mechanism analysis further reveals that labor participation enhances older people’s mental health by shaping positive attitudes toward aging. (3) Notably, the mediating mechanism of positive aging attitudes transforms labor participation from a potential health risk in old age into a key protective factor for mental well-being.

**Conclusion:**

Research findings confirm that Depression Scales serve as crucial indicators for assessing mental health among older adults. Labor participation is a significant psychological protective factor for rural seniors in China. Moderate labor effectively alleviates depressive symptoms and enhances overall mental well-being in this demographic, whereas excessive or relentless labor yields adverse effects. Mechanism analysis indicates that labor participation helps older adults develop positive attitudes toward aging, thereby buffering and mediating the process by which potential health risks are transformed into psychological protective factors. This empirical study provides crucial evidence for policymakers to optimize rural labor force policies and establish intervention systems for active aging.

## 1 Introduction

According to the Seventh National Population Census data, the proportion of rural residents aged 60 and above and above in China reached 23.81%, significantly higher than the 15.82% in urban areas. The proportion of those aged 65 and above stood at 17.72%. Data from the 2023 National Development Bulletin on Aging Affairs indicates that by the end of 2023, China’s population aged 60 and above had grown to 296 million, accounting for 21.1% of the total population. The accelerated pace of population aging has exposed older people to multidimensional challenges in their daily lives.

The “Healthy Aging Plan for the 14th Five-Year Plan Period,” jointly issued by the National Health Commission and 14 other departments in 2022, explicitly identifies the growing prominence of mental health issues as a significant challenge in aging-related work and emphasizes the importance of mental health for older adults. The Blue Book “China Aging Development Report 2024—Mental Health Status of Chinese older adults” indicates that 26.4% of China’s older adults exhibit depressive symptoms, with 6.2% experiencing moderate to severe depression. Depression has become a significant risk factor threatening the physical and mental health, as well as quality of life, among the older adults (1). Exploring protective factors for mental health among older adults is crucial for enhancing their psychological well-being and promoting healthy and active aging. Advancing healthy aging has become a key agenda in contemporary aging-related work, with mental health issues among the older adults receiving widespread attention (2, 3).

Against the backdrop of uneven urban–rural development, rural areas face relative resource scarcity. Rural older adults endure dual pressures from decentralizing social roles and the hollowing out family structures, confronting multiple challenges. Declining incomes weaken economic security, shifts in social roles trigger self-identity crises, and cumulative pressures such as increased disease risks and frequent widowhood intertwine (4). These are compounded by a lack of intergenerational emotional support, scarcity of daily care resources, physical decline, limited access to medical resources, and the outflow of young and middle-aged labor (5). Collectively, these factors exacerbate their vulnerability. These realities collectively accelerate the marginalization of rural seniors’ social roles, while their disembedding from society intensifies psychological burdens. Data from the National Health Commission indicates that only 26.8% of China’s rural older adults maintain adequate mental health (6). Within the coordinated framework of rural revitalization and active aging strategies, exploring systematic intervention pathways for the psychosocial pressures rural seniors face has become critical for advancing the Healthy China Initiative.

At the 15th National Conference on Civil Affairs, President Xi Jinping emphasized that “efforts should be made to promote the implementation of a national strategy for actively coping with population aging” and elevated actively coping with population aging to a national strategy. The State Council of the People’s Republic of China issued the National Plan for the Development of the Aging Career and the Older Service System for the Fourteenth Five-Year Plan, which emphasizes the need to “guide older persons to establish the concept of active health and lifelong development, encourage them to face their old age positively, and give full play to their roles in economic and social development.”

Engagement in economic activities represents the fundamental expression of ‘active aging’ and a critical aspect of social involvement for older individuals. This participation sustains social connections, bolsters self-worth perception, and supports ongoing physical, mental, and cognitive vitality (7). Age discrimination is one of the obstacles encountered in old age, and the participation of older persons in the workforce contributes to breaking down ageist stereotypes, fostering positive attitudes toward aging, and contributing to the creation of a productive society (8).

Against this backdrop, this study utilizes data from the 2020 China older Social Tracking Survey to analyze the impact of labor participation on the mental health of rural older adults individuals and its underlying mechanisms. By clarifying the relationships among labor participation, attitudes toward aging, and the mental health of rural older adults, this study offers new perspectives for related research and attempts to address the following questions: How does labor transition from a survival necessity to an expression of value, and can it yield beneficial effects on the mental health of older people? How can psychological capital compensation be achieved through cognitive restructuring of attitudes toward aging?

## 2 Literature review and research hypothesis

### 2.1 Current situation of labor participation of the older in rural China

Along with the migration of a large number of rural young and middle-aged laborers to cities and towns, the urban–rural gap in population aging has continued to widen, and the structure of the rural resident population has accelerated in its aging. Data from the Seventh National Population Census show that the labor force participation rate of 65-year-olds in rural areas is 29.8 percent, and the proportion of older people over the age of 75 who are still engaged in agriculture is more than 10 percent (9), which confirms the high labor force participation rate of older people in rural areas as a whole. At the institutional level, due to the lack of a clear retirement age and insufficient social security coverage, a large number of older adults at a younger age still need to continue to participate in agricultural production (10, 11). The number of years worked by those employed in agriculture is significantly longer than that of those employed in non-agricultural activities, resulting in the characteristic phenomenon of “endless labor” (12). Rural older persons are not only the target of social care for the aged, but also a special group of people who continue to create health productivity, family function productivity, and labor productivity through labor supply (13). In the context of increasing “hollowing out of rural population,” the young-old is essentially responsible for the core functioning of the agricultural production system. There are a number of factors that influence the labor participation behavior of rural older persons (14). Studies have shown that older persons in rural areas often face inadequate social support compared to their urban counterparts. As a result, withdrawal from the labor market often requires reliance on personal savings or family financial support to meet basic living expenses. This economic vulnerability compels many rural older persons to continue participating in the labor force, primarily to ensure basic survival (15). In addition, the labor participation of rural older people is affected by individual characteristics, and those who are young and in good health have a higher rate of participation in their agricultural production and a higher rate of total labor participation, as well as a longer period of labor supply (16).

### 2.2 The impact of labor force participation on the mental health of older adults

Old age is a central stage in which older people are faced with physical, psychological, and sociocultural transitions that have an impact on their life patterns and state of health, and ultimately on their mental health and quality of life (17). Mental health involves multiple dimensions of an individual’s mental state, emotional expression, behavioral patterns, and interaction with the surrounding environment. Although a uniform definition has yet to emerge from academia, the World Health Organization (WHO) provides a widely accepted view that mental health not only implies the absence of mental illness or psychological disorders, but is also a positive state in which an individual can recognize and utilize his or her abilities to effectively cope with life’s stresses, to work efficiently, and to contribute to the well-being of others. Current academic discussions on factors influencing older adults’ mental health primarily focus on individual factors (18), intergenerational support and community environment (19), as well as social capital and other factors (20).

Under China’s dual system of structure of town and country, there is a long-term imbalance in the allocation of economic and healthcare resources, with urban residents enjoying a relative advantage in the areas of living conditions, lifestyles, and social security (21). Compared to urban older adults who enjoy a relatively complete medical insurance and social support system, rural older adults not only face the dual plight of increasing chronic physical illnesses and frequent mental health problems, but also have to cope with several problems, such as the lack of a medical insurance system and the lack of social support (22). Therefore, conducting relevant social activities becomes an important direction of intervention to alleviate the mental health crisis of this group.

According to social role theory and healthy aging theory, there is a clear duality in the impact of older adults’ labor participation on their health (23, 24). Numerous studies have shown that the accumulation of social capital is negatively correlated with health (25). And labor prolongs the process of social capital accumulation and accelerates the compensatory decline of bodily functions. Continuous and intense physical activity may exacerbate the decline of bodily functions, and this negative effect is more pronounced in rural areas, where the phenomenon of “endless labor” greatly increases the risk of physical and mental exhaustion among older workers (26). In the mental health dimension, labor shows positive value as an important vehicle for social embedding. Based on the Socioemotional Selectivity Theory, sustained productive activity can maintain social network connections (27). Older people’s participation in labor and social interaction activities not only enhances cognitive activity and self-efficacy, but also realizes self-worth, which is not only a manifestation of social function, but also a source of happiness for older people (7, 28). The concept of “productive aging” suggests that rural older persons can contribute to the accumulation of family and social capital through productive practices, creating a unique mental health protection mechanism (29). Empirical studies have shown that moderate participation reduces depressive tendencies and decreases cognitive impairment in old age (30, 31).

From a macro perspective, the labor participation of the aged helps to alleviate the potential constraints on economic growth caused by the structural shortage of the rural labor force; from a micro-individual perspective, labor participation not only expands the social participation channels of the older adults, but also strengthens the sense of self-efficacy through the enhancement of the ability to be economically independent, and in the practice of the value “worthiness of the older adults” The strengthening of dignity and self-identity of the older adults is realized. In this way, they can realize the strengthening of dignity and self-identity in the value practice of “aging well”.

### 2.3 The mediating role of aging attitudes

Attitude toward aging is an individual’s experience and evaluation of the aging process of the self, characterized by strong subjectivity, a more complex psychological structure, and an important measure of the degree of adaptation of older people to the stage of aging (32, 33). Current research on attitudes toward aging presents three distinguishing features: First, most of the existing research focuses on urban or typical regional groups of older people, and the logic and mechanism of aging attitudes among rural older people have not been sufficiently explored. Given the systematic variations in socio-economic structure, healthcare access, and cultural practices between rural and urban settings, these structural disparities can significantly influence the cognitive aging patterns observed in rural older adults (34). Secondly, there is an uncoupled differentiation of influencing factors. Although established studies identify the dual role of endogenous variables, such as gender, age, and one’s health status, and exogenous variables, such as socio-cultural, public resource supply, and social capital stock (8), they fail to adequately reveal the interaction mechanism of internal and external factors.

Maintaining a positive attitude toward aging is a key factor in achieving positive aging. Older adults with positive perceptions of aging view it as an accumulation of experience and wisdom, and can adjust to the aging process more effectively. Empirical studies have shown that positive attitudes toward aging can help improve the mental health of older adults and alleviate depressive symptoms (35). Older adults with positive perceptions of aging usually adapt more smoothly to the process, exhibit better physical and mental health, and participate more actively in social activities, thereby increasing life satisfaction and well-being (36).

Moderate social and labor participation are elements that promote successful aging and contribute to the development of positive attitudes toward aging in older age groups (37). Older people’s participation in productive socio-economic activities can promote positive attitudes toward aging by reshaping cognitive and behavioral patterns of aging (38). On the one hand, labor participation perpetuates the social roles of rural older adults and buffers the anxiety of social disembedding caused by aging. On the other hand, the recognition of economic value and the perception of intergenerational contribution derived from labor behavior can consistently reinforce the psychological construction of positive emotions. The positive effect of labor force participation can reduce the risk of depression among older adults through the cognitive remodeling mechanism of aging attitudes.

### 2.4 Research framework and hypotheses

**Figure 1:**
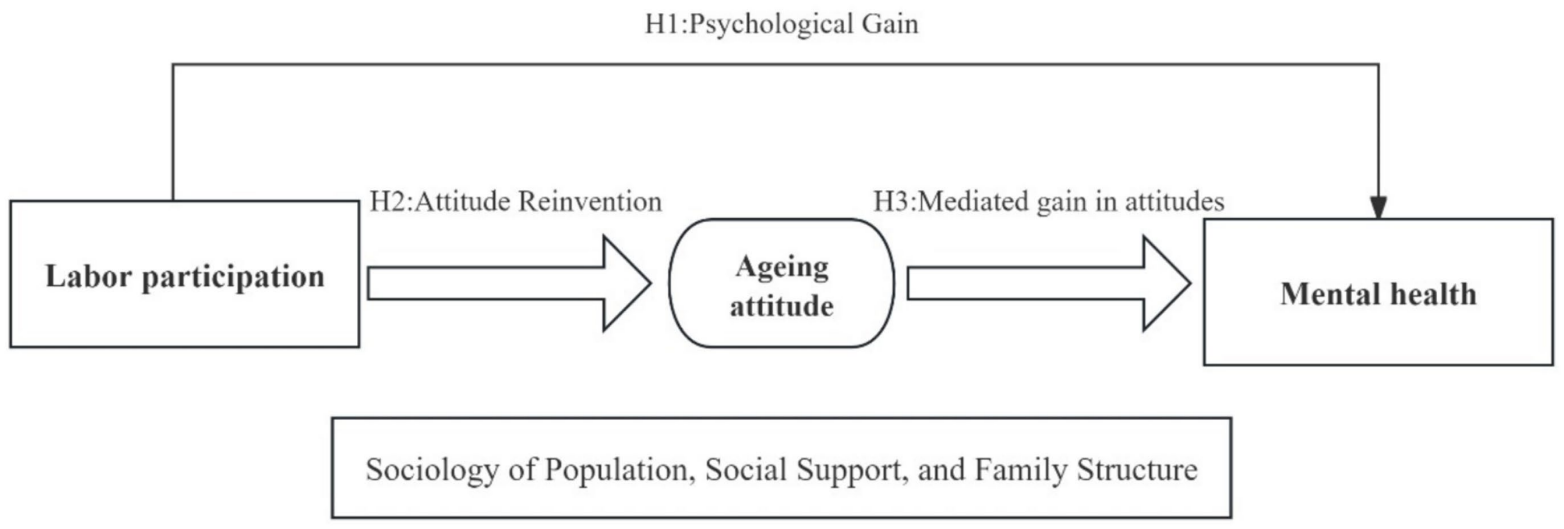
A model of the effects of labor force participation and aging attitudes on the mental health of older adults.

Although existing research has confirmed the independent predictive effects of labor force participation and attitudes toward aging on older adults’ mental health, current analytical frameworks are generally constrained by single-path explanatory logic, failing to effectively reveal the systemic integrated relationship among labor force participation, attitudes toward aging, and mental health. Therefore, to address the limitations of existing studies that predominantly focus on single pathways, this research constructs an integrated “labor participation-attitudes toward aging-mental health” analytical framework (Figure 1). Concentrating on the buffering effect of paid labor on depressive tendencies and its dynamic pathways, this study explores the pivotal role of aging attitudes in labor practices and psychological regulation processes, offering new interpretive dimensions for analyzing the logic of mental health development among rural older adults populations. Based on this framework, the core research questions are articulated using the PECO framework suitable for observational studies:

P (Population): Older residents in rural China (typically aged 60 and above);
E (Exposure): Participation in paid labor;
C (Control): Rural older adults not engaged in paid labor;
(Outcome): Mental health status, with depressive tendencies as a negative indicator.

This framework will provide a new interpretive dimension for analyzing the generative logic of mental health among rural older adults.

Based on the aforementioned research questions and analytical framework, it is imperative to investigate the impact of labor engagement on the mental health of older adults and the mediating role of attitudes toward aging in the relationship between mental health and labor participation, particularly the mechanisms through which these attitudes exert influence. Accordingly, this study proposes and tests the following hypotheses:

*H1*: Labor participation significantly positively affects the mental health of older adults, meaning labor participation alleviates depressive symptoms among older people.

*H2*: Labor participation significantly and positively promotes positive attitudes toward aging.

*H3*: Attitudes toward aging mediate the relationship between labor participation and mental health, meaning labor participation alleviates depressive tendencies by enhancing positive attitudes toward aging, thereby improving mental health.

To test the hypothesis above, this study primarily aims to examine the net effect of labor participation on the mental health of older adults. Secondary objectives include investigating the impact of labor participation on attitudes toward aging and verifying the mediating role of aging attitudes in the “labor participation-mental health” pathway. Furthermore, the robustness of these associations will be assessed by employing methods such as propensity score matching to address potential confounding factors.

## 3 Research design

### 3.1 Data sources and sample selection

This paper selects data from the 2020 China Longitudinal Aging Social Survey (CLASS) organized by Renmin University of China. The survey has conducted detailed research on the basic information, health status, economic status, and psychological feelings of the aged in China, and has a high degree of representativeness and reliability. The study focuses specifically on the older adults because rural areas lag in terms of economic development, social security, and healthcare resources, making the challenges faced by the older adults even more severe. In order to ensure the validity and accuracy of the study, a sample of older adults with agricultural household registration and complete core information was selected, and data with missing or abnormal key variables were excluded. Based on the research theme, cases with missing data on key variables were excluded, and after data processing, 5,117 valid samples were retained.

### 3.2 Selection of model variables

#### 3.2.1 Independent variable

The core explanatory variable, labor force participation, was measured by the question in the CLASS questionnaire, “How often do you currently engage in income-generating work/activities (including agricultural labor, which can also be converted into income)?” Responses were coded as follows: “Almost every day” = 5, “At least once a week = 4,” “At least once a month = 3,” “Several times a year = 2,” and “Not participating = 1”.

#### 3.2.2 Dependent variable

This study draws upon relevant literature (39–41). The Center for Epidemiologic Studies Depression Scale (CES-D) was selected to assess the mental health status of older adults. The CES-D scale has been widely adopted in domestic and international studies to measure mental health among older adults (42). The CLASS questionnaire assessed respondents’ psychological state over the past week through inquiries such as: “Did you feel happy during the past week?” Did you feel lonely in the past week? “Did you feel sad in the past week? “Did you feel happy in the past week? “Did you have poor appetite in the past week? “Did you have poor sleep quality in the past week? “Did you feel physically unwell in the past week? “Did you feel idle in the past week?” “Did you find life enjoyable (interesting things) in the past week?” Nine questions used a three-point Likert scale: “No = 1, Sometimes = 2, Often = 3″. The questionnaire’s Cronbach’s alpha coefficient was 0.864, indicating good reliability. This study reconfigured the options as “No = 0, Sometimes = 1, Often = 2″ and reverse-scored three positive items (“Are you in a good mood?,” “Did you enjoy your life?,” “Was your life full of fun?”). The total score was calculated by summing the scores of each question to form a depression index, where higher values indicate more severe depression and poorer psychological well-being.

#### 3.2.3 Intermediary variable

Following Qiu and Zhou (43) approach, this study measures participants’ attitudes toward aging by assessing their self-perceived aging attitudes. This is done by asking respondents to indicate their agreement with the following statements: “I feel I am already old”; “In my view, growing old is a process of continuous loss”; “After growing old, I find it harder to make new friends”; “Because of my age, I feel excluded.” The responses to these four items were averaged to generate a participant aging attitude variable ranging from 1 (strongly disagree) to 5 (strongly agree).

#### 3.2.4 Control variable

The control variables in this paper are selected based on the relevant literature (35) and cover three main dimensions: socio-demographic characteristics, social support network, and family structure variables. The sociodemographic characteristics variables include gender, age, education level, marital status, older adults’ self-assessed health status, and personal income. The social support network variable encompasses the social support received by older adults, including intergenerational support, kinship ties, and friendships. Family structure variables consisted of the older adult’s residential status and the number of living children.

### 3.3 Methods

This study first conducts a correlation analysis examining the relationships among labor participation, mental health, and attitudes toward aging. It then employs an OLS regression model to estimate and test the impact of labor participation on the mental health of rural older adults. Subsequently, stepwise regression and Bootstrap tests are utilized to validate the mediating effect of aging attitudes. Finally, Propensity Score Matching is applied to conduct endogeneity tests. Data analysis is performed using STATA 18.0 software to verify the hypotheses.

#### 3.3.1 Basic regression model

Since the explanatory variable is the depression score of the aged as a continuous variable, the OLS regression model was used to test whether labor force participation significantly affects the mental health of the older adults, and the baseline measurement model is shown in Equation 1:

$\text{Depression}=\beta_{0}+\beta_{1}\times {\text{Labor}}_{i}+\eta \times X_{i}+\varepsilon_{i}$

Where Depression denotes the level of depression among older adults, Labor represents whether older adults participate in labor, Xi is a control variable, β1 is the intercept, i is a random disturbance term, and are constant term coefficients.

#### 3.3.2 Mediation effects model

To test the mechanism of the role of labor participation in influencing the mental health of older adults, this paper refers to the relevant literature (44) and sets up the following mediation effect model:


(1) $$\text{Depression}=\alpha_{1}+c\times {\text{Labor}}_{i}+\eta \times X_{i}+\varepsilon_{i}$$

(2) $$\text{Attitude}=\alpha_{2}+a\times {\text{Labor}}_{i}+\eta \times X_{i}+\varepsilon_{i}$$

(3) $$\text{Depression}=\alpha_{3}+c\prime \times {\text{Labor}}_{i}+b\times {\text{Attitude}}_{i}+\eta \times X_{i}+\varepsilon_{i}$$

In Equations 1–3, Attitudei represents the mediating variable, i.e., the aging attitude of the aged. a, b, c and c’ are the parameters to be evaluated, respectively, where c represents the total effect of labor force participation on mental health, and c’ represents the direct effect of the aging attitude on mental health, and the product of the coefficients a × b is the indirect effect of labor force participation on mental health (mediated by the aging attitude), and there is the following relationship among the three. And the relationship between the three is as follows:


c=c′+a×b

#### 3.3.3 Tendency score matching model

Numerous factors influence the labor participation behavior of older adults. This study references relevant literature to mitigate endogeneity issues arising from sample self-selection (45, 46) and constructs a propensity score matching model.

We matched working older adults (treatment group) with non-working older adults possessing identical characteristics (control group). Using observable variables as controls, we constructed a set of covariates to estimate conditional probability fits for labor participation and mental health—the “propensity score.” The propensity score (PS) calculates the probability of an individual sample entering the treatment group based on the existing covariate set, employing a Logit model expressed as follows:


$$\mathrm{PS}(X_{i})=P_{r}(D_{i}=1|X_{i})=\frac{\exp(\beta_{0}+\beta \prime X_{i})}{1+\exp(\beta_{0}+\beta \prime X_{i})}$$

After estimating propensity scores, treatment and control groups are matched based on propensity score values. The matching quality is assessed through standard support domain tests and balance tests.

## 4 Results

### 4.1 Descriptive statistics

According to the descriptive statistics in Table 1, the distribution of key variables in this study is as follows. The mean score for the dependent variable, depression severity, measured using the CES-D scale, was 7.019 (SD = 3.212), ranging from 0 to 17. Higher scores indicate more severe depressive symptoms, suggesting that the sample group’s mental health level falls within the lower-middle range, with significant inter-individual variation. The mean value for the explanatory variable labor participation was 2.252, measured on a 5-point frequency scale ranging from “not at all” to “almost daily.” The mean approached the “a few times a year” level, indicating overall low labor participation frequency among older people in the sample. However, significant differences existed in participation levels across individuals, reflecting heterogeneity in labor behavior. The mean value for the mediating variable, attitude toward aging, was 2.891. Measured on a 1–5 scale, higher values indicate a more positive attitude. The current mean falls in the upper-middle range, suggesting that the sample group holds a generally positive attitude toward aging, with moderate variation among individuals.

**Table 1:** Variable settings and descriptive analysis.

Variables		Description of variables	Mean	SD	Min	Max
Independent variable	Labor Participation	1 = No participation; 2 = Several times a year; 3 = At least once a month; 4 = At least once a week; 5 = Almost every day	2.252	1.651	1	5
Dependent variable	Depression Level	Depression CES-D scale scores summed	7.019	3.212	0	17
Mediator	Aging attitude	1–5 = totally negative-totally positive	2.891	0.881	1	5
Control variables	Gender	0 = Female; 1 = Male	0.523	0.500	0	1
	Age	Actual age	71.551	6.332	60	98
	Marital status	0 = No spouse (widowed, divorced, unmarried); 1 = Married with a spouse	0.743	0.437	0	1
	Educational attainment	0 = Middle school and below; 1 = High school and above	0.039	0.195	0	1
	Self-assessment	0 = Unsatisfied 1 = Fair; 2 = Very satisfied	1.331	0.707	0	2
	Income	The logarithmic sum of an individual’s income for the last 12 months and the amount of benefits received from the various social security programs is obtained by adding 1 to the sum.	4.768	2.116	0	11.609
	Children’s Financial Support	0 = None; 1 = Yes	0.925	0.263	0	1
	Children’s Household Support	1–5 Almost None-Almost Everyday	2.850	1.175	1	5
	Relatives’ Contacts	1–5 = None-9 and Above	7.346	2.696	0	15
	Friends’ Contacts	1–5 = None-9 and above	6.666	3.104	0	15
	Living with status	0 = No; 1 = Yes	0.885	0.319	0	1
	Number of living children	Total number of living children of respondent	2.750	1.245	0	9

Regarding control variables, the gender distribution was balanced, with males accounting for 52.3% and females 47.7%. The age range spanned from 60 to 98 years old, with seniors aged 80 and above accounting for 18.7%. Regarding marital status, 74.3% of older people were married, while 25.7% were unmarried. Educational attainment showed that 96.07% had completed junior high school or below, with only 3.93% having received senior high school education or higher. This educational gap may limit rural seniors’ access to health resources and contribute to weaker psychological coping abilities. The mean self-rated health status was 1.33, falling between “dissatisfied” and “fair,” indicating a relatively negative overall health perception among the sample. 52.98% of older people considered their health poor, which correlates with the low accessibility of medical resources in rural areas. Significant income disparities indicate that most older adults have lower incomes, highlighting their economic vulnerability. Financial support from children is common, with approximately 92.5% receiving such assistance. The average score for household chores support from children is 2.85, indicating moderate frequency of such support. Both findings reflect the traditional role of the family in elder care. Regarding social interaction, contact with relatives and friends is relatively frequent, with average scores of 7.35 and 6.67, respectively. Regarding living arrangements, 88.5% of older people live with others. Most older adults have two or more children, which is consistent with the realities of rural areas.

In summary, the overall characteristics of the sample indicate the multiple vulnerabilities that older adults are facing (47), aligning with the actual circumstances of the rural older cohort. Together, these structural factors constitute mental health risks for the aged, providing a realistic context for subsequent analysis.

### 4.2 Correlation analysis

This study examined the intrinsic relationships among three core variables—depression severity, labor participation frequency, and attitudes toward aging—through Pearson correlation analysis. As shown in Table 2, statistically significant correlations exist among all variables, preliminarily revealing the basic framework of the psychological health impact mechanism among workers.

**Table 2:** Correlation analysis of mental health, attitudes toward aging, and labor participation.

Variables	Depression level	Labor participation	Aging attitudes
Depression level	1.00		
Labor participation	−0.112*	1.00	
Aging attitudes	−0.199*	0.126*	1.00

*p < 0.1, **p < 0.05, ***p < 0.01, same below.

Depression levels negatively correlated with labor participation frequency (*r* = −0.112, *p* < 0.05). Older adults with higher labor participation exhibited lower depressive symptoms and better psychological well-being. Therefore, encouraging and supporting older adults to engage in age-appropriate labor can effectively improve their mental health, though the underlying mechanisms warrant further investigation.

Mental health was negatively correlated with attitudes toward aging (*r* = −0.199, *p* < 0.05). Individuals with more positive attitudes toward aging exhibited better mental health. Positive aging attitudes manifest as acceptance of aging, optimistic expectations for later life, and affirmation of personal value. Such positive cognition alleviates psychological stress from physical decline and social role transitions, enhancing individuals’ psychological adaptability. In rural settings, older adults with positive aging attitudes better adapt to age-related changes and maintain sound psychological states.

Labor participation frequency positively correlates with aging attitudes (*r* = 0.126, *p* < 0.05). Older adults with higher levels of labor participation exhibit more positive attitudes toward aging. Labor participation provides opportunities for social engagement, helping maintain a sense of social connectedness and self-worth. Through work, older adults can demonstrate their capabilities and value, countering age discrimination and social exclusion, thereby fostering a more positive identity in old age. In rural areas, labor participation is a crucial pathway for older adults to maintain social activity, playing a promotional role in shaping positive attitudes toward aging.

In summary, the correlations among variables indicate that labor participation may indirectly influence mental health by affecting attitudes toward aging. On one hand, labor participation promotes the formation of positive aging attitudes; on the other hand, positive aging attitudes contribute to maintaining mental health. Labor participation exerts a dual impact on mental health: a direct pathway manifests as a negative influence, while an indirect pathway yields a positive effect through aging attitudes. This complex mechanism reflects the multidimensional psychological effects of labor participation among rural older adults.

### 4.3 Regression analysis

This study empirically examined how labor participation influences mental health by constructing stepwise regression models. As shown in Table 3, Models 1 to 3 sequentially incorporated linear terms, nonlinear terms, and a series of control variables. The results revealed a significant inverted U-shaped relationship between labor participation and mental health.

**Table 3:** Basic regression results.

Variables	Model 1	Model 2	Model 3
Labor participation	−0.219^***^	1.235^***^	1.530^***^
	(−8.00)	(6.29)	(8.00)
The square of labor participation		−0.256^***^	−0.291^***^
		(−7.40)	(−8.67)
Gender			−0.189^*^
			(−2.18)
Age			0.036^***^
			(4.57)
Marital status			−0.190
			(−1.57)
Educational attainment			−1.088^***^
			(−4.74)
Self-assessment			−1.000^***^
			(−15.98)
Income			0.009
			(0.37)
Children’s financial support			−0.426^**^
			(−2.74)
Children’s household support			−0.112^**^
			(−3.02)
Relatives’ contacts			0.051^*^
			(2.35)
Friends’ contacts			−0.127^***^
			(−6.54)
Living with status			−0.557^***^
			(−3.65)
Number of living children			−0.067
			(−1.77)
_cons	7.507^***^	6.225^***^	6.690^***^
	(97.63)	(32.93)	(10.90)
*N*	5,117	5,117	5,117
*R*^2^	0.01	0.02	0.11
Adj. *R*^2^	0.01	0.02	0.11

Model 1 examined only the linear relationship between labor participation and mental health among older adults. Results indicate that labor participation alleviates depressive symptoms and improves mental health (*β* = −0.219, *p* < 0.001), confirming Hypothesis 1. In Model 2, introducing the quadratic term for labor participation frequency transformed the linear coefficient into a significant positive value (*β* = 1.235, *p* < 0.001). In contrast, the quadratic coefficient remained significantly negative (*β* = −0.291, *p* < 0.001), consistent with an inverted U-shaped relationship. Model 3 further controlled for individual characteristics, social support, and family structure variables. The inverted U-shaped relationship remained robust. The coefficient for the linear term of labor participation increased to 1.530, while the coefficient for the quadratic term expanded to −0.291, both maintaining significance at the 0.1% level.

Additionally, regarding individual characteristics, gender, age, and educational attainment showed significant correlations with older adults’ mental health. This indicates that compared to other groups, older women with lower educational levels and poorer physical health exhibit lower psychological capital and are more prone to depressive moods. This situation may be linked to the multiple challenges rural older adults face, including relatively underdeveloped educational and medical resources and prolonged social isolation. Regarding intergenerational support, financial assistance, and household help provided by children contribute to psychological gains among rural older adults, alleviating depressive symptoms. In terms of social networks, maintaining a certain level of friendship connections helps mitigate depression and improve mental health. In contrast, frequent contact with relatives tends to exacerbate depressive feelings among older people.

### 4.4 Mediation effect test

The mediating effect analysis was conducted using the stepwise method with labor participation as the independent variable, depression score as the dependent variable, and aging attitude as the mediator variable (48). Since the results of the analysis of Model 1 in the previous section have confirmed the significant effect of labor participation on depression score, it has the prerequisites to test the mediating effect by the stepwise regression method.

Table 4 shows the results of the regression analysis. Among them, model 3 is the test of the independent variable labor participation on the mediator variable aging attitude, and the results show that labor participation is positive and significant at the 1% level on the aging attitude of rural older adults, which explains that labor participation does help rural older adults to form positive aging attitudes, and thus verifies Hypothesis 2. Model 4 is the test of the mediator variable, aging attitude, on the dependent variable depression, and the results show that labor participation also has a significant effect on depression when controlling for aging attitude, as well as the rest of the variables. Attitudes and the rest of the variables, labor participation, also have a significant effect on depression. That is, the higher the labor participation of the aged, the more positive their aging attitudes, which in turn reduces depression and improves mental health, confirming the mediating role of aging attitudes and therefore verifying the research hypothesis 3.

**Table 4:** Results of the mediation effect test based on aging attitudes.

Variables	Model 4	Model 5
	Aging attitude	Depression level
Labor participation	0.042***	−0.101***
	(5.50)	(−3.71)
Aging attitudes		−0.614***
		(−12.04)
Control variables	YES	YES
_cons	3.377***	10.05***
	(19.31)	(16.12)
*N*	5,117	5,117
*R*^2^	0.05	0.13
Adj. *R*^2^	0.05	0.12

To further test the mediating mechanism that aging attitudes play in the relationship between labor force participation and mental health in old age, the test was conducted using Hayes’ Bootstrap method, which verifies the presence or absence of a mediating effect by determining whether or not the Bia (Bias-corrected) 95% confidence interval contains zero.

As shown in Table 5, after conducting 1,000 bootstrap samples, the results indicate that the direct effect of labor participation on depression is −0.101, with a 95% CI [−0.155, −0.048] that does not include zero. The indirect effect mediated through aging attitudes is −0.026, with a 95% CI [−0.035, −0.016] that does not include zero. Both effects are statistically significant at the 1% level. The mediation effect accounted for 20.22% of the variance, indicating that attitudes toward aging significantly mediated the relationship between labor participation and depression severity. The mediation path was established, further validating the intrinsic pathway of “behavioral practice-attitude reshaping-psychological gain” in the older adults influenced by labor participation.

**Table 5:** Moderated mediation model.

Effect component	Effect	SE	P>|z|	95%CI	Effect ratio %
Total effect	−0.127	0.051	0.000	[−0.713,-0.514]	100
Labor participation → Mental health	−0.101	0.027	0.003	[−0.155,-0.048]	79.78
Labor Participation → Aging Attitudes → Mental health	−0.026	0.005	0.000	[−0.035,-0.016]	20.22

### 4.5 Endogeneity test

Given that regression coefficients in OLS models may be subject to sample selection bias—meaning that participation in labor among older people is not a random event but a “selective” outcome influenced by factors such as physical health, social connections, or socioeconomic status due to the existence of health inequality effects— Performing regression analysis on non-random sample data inevitably leads to biased model estimates. To obtain more reliable and effective parameter estimates, the endogeneity issue caused by sample selection bias must be addressed (49). Therefore, to resolve endogeneity concerns, this study employs Propensity Score Matching (PSM) to reassess labor participation’s impact on older adults’ mental health.

Table 6 presents the results of propensity score matching estimates. Model 6 represents the OLS regression without propensity score matching, while Models 7 to 9 employ regression equations with different propensity score weighting strategies. Specifically, Model 7 applies the nearest neighbor matching method, Model 8 uses the kernel matching method, and Model 9 employs the radius matching method. The regression results indicate that regardless of the matching method—nearest neighbor, kernel, or radius—labor participation exerts a significant adverse effect on depression levels, thereby enhancing mental health. The correlation coefficients range from −0.194 to −0.408, all statistically significant at the 5% or 1%. This conclusion remains robust across different matching methods, confirming that labor participation alleviates depressive tendencies and improves mental health.

**Table 6:** Propensity score matching estimation results.

Variables	Model 6	Model 7	Model 8	Model 9
	OLS	PSM 1	PSM 2	PSM 3
Labor participation	−0.324^**^	−0.408**	−0.194*	−0.194*
	(−3.24)	(−3.24)	(−2.06)	(−2.06)
Control variables	YES	YES	YES	YES
_cons	7.534***	8.054***	8.620***	8.620***
	(12.71)	(9.34)	(12.75)	(12.75)
*N*	5,117	3,229	5,111	5,111
*R*^2^	0.10	0.08	0.08	0.08
Adj. $R^{2}$	0.09	0.08	0.08	0.08

Table 7 presents the average treatment effects of labor participation on the mental health of older adults. Results indicate that older adults engaged in labor activities scored significantly lower on depression measures—ranging from 0.196 to 0.536 points—compared to their non-working counterparts, suggesting superior mental health among active participants. This aligns with the linear regression findings discussed earlier, confirming that labor participation effectively mitigates depressive tendencies among older adults, enhancing their mental well-being.

**Table 7:** Tendency score matching average processing effect.

Matching scheme	Participating labor group (1)	Non-participating labor group (2)	ATT = (1)–(2)	SD	*t*
Depression level					
Nearest neighbor matching	6.696	6.891	−0.195	0.144	−2.062**
Nuclear matching	6.694	7.230	−0.536	2.036	−0.052
Radius matching	6.696	7.232	−0.536	1.619	2.741**

### 4.6 Robustness

In order to ensure the robustness of the benchmark regression results, this paper further conducts robustness tests by setting up replacement variables and replacement models (see Table 8).

**Table 8:** Robustness tests.

Variables	Model 10	Model 11
	Replace the explained variable	Replacement metrology model
Labor participation	0.016*	−0.127***
	(2.20)	(−4.67)
Control variables	YES	YES
_cons	3.022***	2.551***
	(17.49)	(222.517)
*N*	5,117	5,117
*R*^2^/Pseudo *R*^2^	0.110	0.020

#### 4.6.1 Substitution of variables

Model 10 replaces the dependent variable “depression level” with “life satisfaction” in the regression analysis. The life satisfaction variable originates from the CLASS questionnaire item: “Overall, are you satisfied with your current life?” Responses ranging from “Very dissatisfied” to “Very satisfied” were coded 1 to 5, respectively. Empirical results indicate that labor participation significantly positively affects life satisfaction at the 5% statistical significance level. This finding not only aligns with the main model’s conclusion that “labor participation reduces depression levels” but also substantiates the core proposition of this study: “labor participation exerts a protective effect on mental health”.

#### 4.6.2 Substitution of models

Model 11 employs an ordered Tobit model for estimation. The results indicate that the regression coefficient for labor force participation on depression levels among older people is −0.127. This finding aligns with the previous analysis, demonstrating that the research conclusions remain robust even after changing the econometric model.

## 5 Discussion

Traditional eldercare models face pressure to restructure against the backdrop of agricultural modernization and shifting family structures. As rural aging intensifies, older adults confront dual challenges of livelihood security and psychological adaptation. This study focuses on the intervention effects of labor participation on mental health. Empirical analysis reveals that labor participation significantly delays the deterioration of depressive symptoms among older people, consistent with existing research findings (7, 50). However, this study further identifies that this psychological benefit exhibits a “threshold effect”. The mediation model confirms that attitudes toward aging play a crucial mediating role between labor participation and mental health—labor behavior fosters positive aging evaluations among older adults by enhancing self-worth recognition and reducing perceptions of age discrimination. This finding validates the concept-shaping function of older adults’ labor participation (39), complementing existing frameworks that simplistically categorize labor participation as purely economic behavior. It further reveals the empowering effect of labor as a form of social engagement on psychological well-being. By systematically examining the mediating pathways of aging attitudes, this study elucidates the underlying psychological mechanisms through which labor participation influences mental health, thereby deepening our understanding of theoretical frameworks for social participation among older adults.

First, labor activities themselves directly alleviate depressive symptoms by maintaining social functions. Activity theory suggests that by participating in various types of social activities, older persons can not only integrate into society, establish new social networks and acquire new roles, but also effectively improve the emotional distress caused by the disruption of social roles in old age and help older persons to reacquaint themselves with their selves, thereby maintaining the psychological well-being of older persons (51, 52). Participation in paid labor not only relieves livelihood pressure on older adults but also produces significant psychological empowerment effects, which are consistent with the findings of previous scholars and further confirm the positive role of labor participation in promoting older adults’ mental health (53). In rural communities, where the hollowing out of the population is a significant feature, labor participation essentially constitutes a social field for the reactivation of the human capital of the aged. By embedding collective farming, mutual aid and cooperation and other forms of localized labor, rural older persons have been able to reconstruct their social relationship networks, and as the participation rate in other social activities, such as collective farming, continues to increase, it effectively buffers the psychological stress caused by loneliness and marginalization of rural older persons, reduces the occurrence of depression and improves their mental health status (54).

Second, labor participation exhibits an inverted U-shaped relationship with the mental health of rural older adults, meaning that “endless” and excessive labor can harm their psychological well-being. Research by Huang and Lü (55) confirms that continued labor participation negatively impacts older adults’ life satisfaction. This study further expands this understanding by establishing the adverse effects of excessive labor and precisely identifying the critical threshold where labor participation shifts from positive to negative. From the perspective of active aging, we should neither completely dismiss the positive significance of labor participation for older adults’ mental health nor overlook the potential risks of overwork. The ideal policy design should focus on helping older adults find the “optimal range” for labor participation, leveraging its psychological benefits while preventing them from overworking.

Third, labor practices indirectly alleviate depressive symptoms by fostering a positive perception of aging. A measurement framework based on the theory of successful aging (56) revealed that older adults who consistently engage in productive work have significantly higher levels of aging self-efficacy and significantly lower levels of internalization of their aging stereotypes. The path of this transformation is broadly characterized by the redefinition of one’s social role through labor participation, the realization of one’s self-worth, and the enhancement of mental health mechanisms. Specifically, the role-reconstruction effect generated by the labor field enabled participants to gain respect through productive contributions, which in turn strengthened self-efficacy, optimistic tendencies, hope, and stress resistance. This accumulation of psychological resources significantly buffers the psychological risks associated with the perception of age discrimination. More importantly, the mutual-help social networks generated by labor participation form an informal support system that creates spatial spillovers of mental health promotion effects, especially in rural areas with high rates of labor exodus.

In addition, this study found significant individual characteristics of mental health risk among rural older adults. Women, older adults, unmarried or widowed, less educated, individuals with poorer physical health, and those with lower personal incomes were more likely to be depressed (58). Older adults who lacked intergenerational support, lacked emotional support from friends, and lived alone had a higher tendency to depression and poorer mental health. In addition, studies have found that high-frequency relative interactions may increase the risk of depression, which may be related to the existence of the “paradox of obligatory contact,” whereby structured family interactions may exacerbate psychological distress in older adults (57). There may be hidden costs of relational stress in traditional kinship networks, the underlying mechanisms of which urgently need to be deepened through mixed research methods.

## 6 Conclusion

First, based on the cultural roots and relationship networks of local communities, innovative modes of social participation for the aged have been developed to provide age-friendly jobs that combine “productive participation” with “developmental participation.” Traditional farming experience and local culture inheritance have been incorporated into the list of services, such as the establishment of “farming instructor” positions to encourage experienced rural elders to provide planting guidance to new farmers returning to their hometowns, which not only activates the old age dividend but also promotes intergenerational knowledge transfer. The transformation of the identity of older groups from “experience holders” to “rural think tanks” has been realized, providing a new paradigm for the governance of aging.

Second, a support system centered on flexible work arrangements, labor time guidance, and mental health services should be established to foster a positive synergy between labor participation and mental well-being among older adults. Based on this study’s revelation of the inverted U-shaped impact mechanism of labor participation on mental health, the core principle lies in helping older adults regulate their labor participation intensity within the optimal range that maximizes marginal benefits. Specifically, flexible work arrangements must fully accommodate the physiological characteristics and varying health conditions of the older adults, granting them autonomy to dynamically adjust work pace and duration according to their energy levels, thereby avoiding rigid, one-size-fits-all constraints. Priority should be given to promoting work models aligned with older people’s physiological and health characteristics, such as segmented work schedules, seasonal employment patterns, and remote work. This approach ensures their social engagement while reserving sufficient time for rest and physical/mental recovery, thereby achieving synergistic enhancement of labor value and mental well-being.

Third, deepening the localization and innovation of the “time bank” mutual-help older adults care mechanism, and establishing an older care service system based on social networks of acquaintances in rural areas. Strengthen investment in the construction of mutual-help pension systems and education for the older adults in rural areas, and set up a special fund to support the construction of a pension service system based on villagers’ committees as the mainstay of the mutual-help network of neighbors, equipped with the necessary facilities and service personnel, to provide the aged with services such as health counseling, day-to-day care, and emergency relief. This kind of innovative practice, with acquaintance capital as the link and cultural identity as the lubricant, helps to reconstruct the dialectical relationship between “active aging” and “care for the aged”.

Fourth, it actively guides older persons to establish the concept of lifelong learning, encourages them to participate in lifelong education programs, continuously improves their self-cultivation and personal abilities, and advocates an optimistic attitude toward life. Through the design of aging-friendly curricula, older people are helped to acquire digital skills and knowledge of social change, so as to alleviate the decline in self-efficacy triggered by technological dislocation. The learning process is embedded in community service networks, and activities such as volunteer teaching and skill sharing are used to reshape older people’s practical knowledge of “active aging” and the psychological buffer against age discrimination, so that older people can realize the identity leap from passive adaptation to active construction in social participation.

### 6.1 Limitations

Although this paper builds upon existing research by conducting more in-depth exploration and refinement in research content and methodology, certain areas still warrant improvement. The time lag in the data samples imposes certain limitations on the research findings. Future studies could incorporate additional interviews and longitudinal cohort studies, employ random sampling methods, balance various factors, expand sample sizes, and conduct more in-depth investigations into the relationship between labor participation and mental health.

## Data Availability

Publicly available datasets were analyzed in this study. This data can be found at: 2020 China Longitudinal Survey of the Aging Society (CLASS).
